# Microstructural Evolution and Internal Hydrogen Content of Ultra-High-Strength Automotive Steels During Two Typical Industrial Production Flows

**DOI:** 10.3390/ma18092034

**Published:** 2025-04-29

**Authors:** Zhiyuan Chang, Jingjing Yin, Long Li, Xingzhao Chen, Xinyi Ruan, Liangyun Lan

**Affiliations:** 1 State Key Laboratory of Vanadium and Titanium Resources Comprehensive Utilization, Pangang Group Research Institute Co., Ltd., Panzhihua 617000, China; yjyyjj@pzhsteel.com.cn (J.Y.);; 2 School of Mechanical Engineering and Automation, Northeastern University, Shenyang 110819, China; 15100295751@163.com (X.C.); 18225122538@163.com (X.R.)

**Keywords:** hydrogen content, hot stamping, microstructure, Al-Si coating, hydrogen uptake

## Abstract

Hot stamping is a promising method to manufacture ultra-high-strength automotive steel components with high dimension accuracy. In this work, two actual industrial production flows (with and without Al-Si hot dipping) were investigated to reveal their microstructural evolution and hydrogen content at different production steps. Meanwhile, the variations in composition and phase structures of the Al-Si coating layer were studied in terms of energy-dispersive spectrometry and electron backscattering diffraction techniques. The results showed that the microstructure at the steel substrate changed from the pancake-shaped pearlite and ferrite, degenerated pearlite and annealed ferrite, lath martensite, and then tempered martensite with the progress of the production steps, which was not affected by the Al-Si hot dipping. The final coating layer exhibited a multi-sublayer structure with the alternative distribution of FeAl and Fe_2_Al_5_, which contained many microcracks on the brittle phase Fe_2_Al_5_. The Al-Si-coated specimens always had higher hydrogen content than the bare steel specimens because of the hydrogen generation at the hot stamping stage and hydrogen absorption during the hot-dip aluminizing stage.

## 1. Introduction

Automotive lightweighting is an effective method to reduce greenhouse gas emissions for the use of vehicles. Ultra-high-strength steels (e.g., 2 GPa tensile strength) have been developed to fulfill this technique in consideration of crash safety [1,2]. Compared to cold forming, the hot stamping process is a promising alternative for producing automotive structural components with high mechanical strength [3]. Importantly, it can significantly reduce the spring back and improve the dimensional accuracy, especially for complex parts [2,4,5].

The industrial production line of press hardening steels (PHSs) normally includes a series of successive processing steps, e.g., hot rolling, cold rolling, continuous annealing (with or without hot dipping), hot stamping, and bake hardening. Although hot stamping is a basic step to obtain the ultra-high-strength parts through martensite transformation strengthening [6,7,8], other steps are also involved in microstructural evolution, and some microstructure characters can be inherited step by step to influence the final mechanical properties. For instance, heat treatment at the continuous annealing step determines the initial microstructure of hot stamping, which influences austenitizing at the hot stamping stage, like carbide dissolution kinetics and austenite grain size. Jarvinen et al. [9] used four different annealing treatments to obtain different initial microstructures and investigated their effect on the final properties of press-hardened 22MnB5 steels. Their results showed that the relatively uniform distribution of pearlite or cementite in the ferritic matrix can lead to the smallest prior austenite grain size. In a sense, it is paramount that all these processing steps that are involved with microstructural evolution should be accounted for to optimize the final properties of the product.

Delayed fracture or hydrogen embrittlement (HE) is also a challenge for the manufacturing and application of such ultra-high-strength steels, since higher strength, higher susceptibility to the HE [10,11,12,13], and atomic hydrogen might be produced during the processing steps, e.g., pickling, painting, and hot stamping [10,14,15,16]. It is known that the Al-Si-coated steel sheet usually contains higher hydrogen content than the bare steel sheet after hot stamping, because the dew point in the furnace is not controlled on the industrial production line, and the molten Al can react with the water vapor to produce the hydrogen atoms [14,15,17,18,19,20]. Cho et al. [18] found that the Al-Si-coated steel has a greater sensitivity to both the hydrogen uptake and the resultant embrittlement than the uncoated and galvanized steels. Recently, Ostwald et al. [19] also substantiated the hydrogen absorption of ultra-high-strength Al-Si-coated 22MnB5 steels using different humidified process gases during simulation hot stamping. Krid et al. [20] analyzed the hydrogen trapping and diffusion in aluminized press-hardened steels that were produced using controlled wet gas in a muffle furnace and showed that the hydrogen was trapped in the steel substrate, and the coating layer had a barrier effect on the hydrogen degassing at ambient temperature.

Delayed fracture has been occasionally reported in actual industrial productions in only one state or a certain assembly line for such ultra-high-strength aluminized steels [16,21]. Obviously, these studies always focus on the hot stamping step using the processing simulation method, as it is the most important step during the industrial production chain, and the hydrogen uptake occurs notably at this step for the aluminized steels. However, as mentioned above, there is a long industrial flow chain for automotive steel production. It is still unclear how the hydrogen content evolves after the hot stamping, and whether the hydrogen might be introduced into the steel matrix at any other steps. In addition, the coating layer of the aluminized steel sheet not only determines the hydrogen uptake and degassing but also influences the bending property due to the nature of the brittleness of some intermetallic phases that were formed during the coating reaction [17,22].

The hydrogen content and microstructural evolution throughout the industrial chain directly determine the collision safety performance and service life of products, yet existing literature to date has not fully elucidated these problems. In current industrial production, PHS components still occasionally exhibit hydrogen-induced delayed cracking. Thus, the main goal of this work is to investigate the microstructural evolution (especially the structure of the coating layer) and hydrogen content based on the actual industrial production flows for ultra-high-strength PHS, which can offer some basic knowledge to optimize the processing parameters and improve the resistance to delayed fracture.

## 2. Materials and Methods

**Figure 1:**
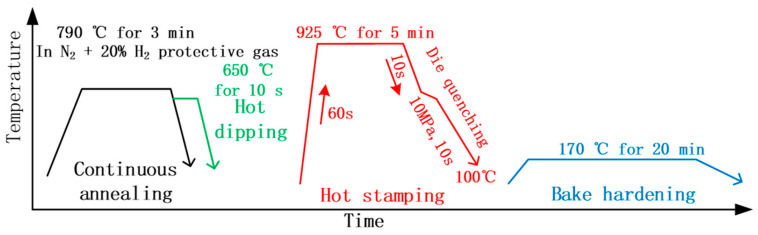
Schematic of heat treatment for both actual industrial production lines.

The actual industrial production line of our objective was located at the Pan steel plant in Xichang City, Sichuan Province, China, and the chemical compositions of the ultra-high-strength automotive steel coil that was produced on this production line were 0.32~0.36 C, 1.6 (Mn + Si), 0.05 (Ti + Nb), 0.03~0.15 V, 0.003 B (PHS2000), balanced by Fe (wt.%). The average thickness of the steel coil was about 1.5 mm, and the final tensile strength reached to 2 GPa with good ductility. The details of mechanical properties were reported elsewhere [23]. Figure 1 schematically shows the heat treatment schedules with and without the hot dipping step for two actual industrial production flows. One process included continuous annealing (790 °C for 3 min in a protective gas of N_2_ + 20% H_2_). The hot stamping process is as follows: Heating the material from room temperature to 925 °C using 60 s, maintaining at this temperature for 5 min. Then, transferring the specimen to the mold for 10 s, followed by applying 10 MPa press pressure, holding for 10 s to ensure the sheet temperature drops below 100 °C (die quenching), and bake hardening (170 °C for 20 min), which was named as the bare steel process due to uncoating. By contrast, the hot dipping (650 °C for about 10 s in a melting solution of Al − 10% Si) was added intermediately after the continuous annealing for the other process, which was referred as the Al-Si coating process. Note that the dew points of the furnace at the hot stamping were not controlled for both industrial processes.

The metallographic specimens were directly extracted from the steel coil at each industrial step and then subjected to conventional preparation, such as grinding, polishing, and etching. The microstructures were observed using optical microscope. A field-emission scanning electron microscope (SEM) equipped with electron backscattering diffraction (EBSD) was applied to identify the crystallographic characters of substrate microstructure and coating layer at different processing steps after these selected samples were electropolished again in a solution of 10% HClO_4_ and 90% ethanol. The energy-dispersive spectrometry (EDS) attached to the SEM was used to confirm the change in chemical compositions of the Al-Si coating layer during hot stamping. X-ray diffraction (XRD) experiments were conducted on a Rigaku SmartLab 9 kW X-ray diffractometer (scan rate 2°/min, Cu-Kα radiation, Tokyo, Japan) to analyze the phases of Al-Si coating in hot-dipped and hot-stamped conditions. The intricate substructures of martensite were observed using a field emission transmission electron microscope (TEM) after thin foils were extracted from hot-stamped and bake-hardened steel plates, and they were subjected to the TEM sample preparation. For matrix microstructure characterization, all samples were extracted from the received steel sheet randomly, because the matrix microstructure was fully martensitic. However, to statistically analyze the thickness of the coating layer, three metallographic samples were taken from the head, middle, and tail of the coil, and at least five random fields of view were checked to fix the coating layer thickness for each metallographic sample.

The tensile properties were measured on the specimens with a gauge length of 50 mm and a gauge width of 12.5 mm, according to the ASTM-E8 standard [24]. Tensile specimens were machined with the longitudinal axis parallel to the hot rolling direction, and two specimens were used for each condition. The tensile test was performed at room temperature with a crosshead displacement speed of 2 mm/min. The size of the three-point bending specimens was 60 mm × 60 mm × 1.5 mm (rectangular). Three-point bending tests were conducted on a universal testing machine at room temperature. Three-point bending experiments were measured, according to the VDA 238-100 standard [25]. The punch speed was 20 mm/min, and the punch radius was 0.4 mm. The three-point bending angle was calculated based on the punch displacement. Tensile tests and three-point bending were carried out using a Instron 50 kN testing machine (Norwood, MA, USA), and three specimens after removing the iron oxide layer were used for each condition. For hydrogen content analysis, after each production step the samples (15 by 6 mm in size with through-thickness) were cut from the steel sheet as fast as possible (within two minutes) and kept in a liquid nitrogen container to avoid interior hydrogen degassing out. The melt extraction method was employed using a LECO ONH836 hydrogen determinator (St. Joseph, MI, USA, detection resolution 0.01 ppm, measurement range 0.5–8 ppm) to obtain the total hydrogen concentration of all specimens after they were taken out from the liquid nitrogen (to minimize or prevent diffusible hydrogen escape to the greatest extent practicable) and ground carefully with sandpapers to remove the surface oxide scale, especially for the bare steel specimens, followed by ethanol rinsing. Each condition was tested five times, and the average hydrogen content is reported below to show its evolution with the process flow.

## 3. Results and Discussion

### 3.1. Microstructure and Mechanical Properties

**Figure 2:**
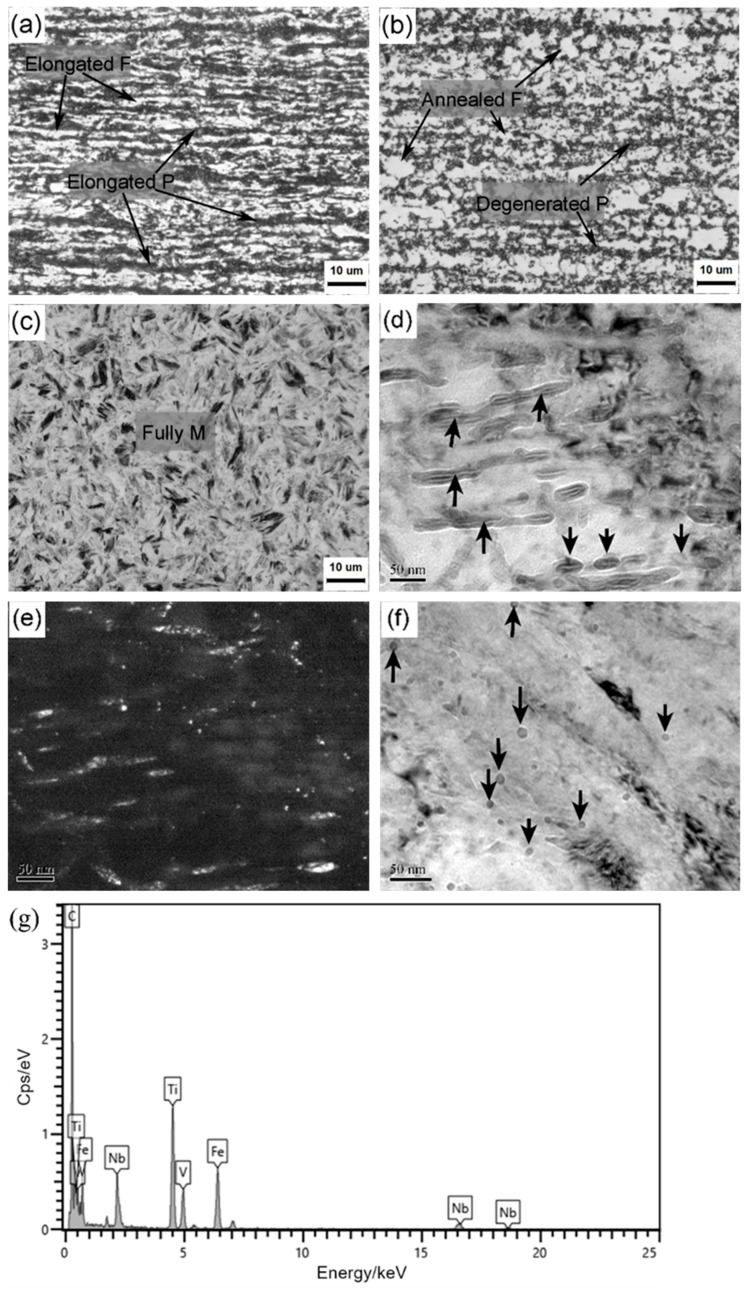
Optical images showing microstructural evolution after subjecting to different processing stages: (**a**) cold rolling, (**b**) continuous annealing, and (**c**) hot stamping, and TEM images showing the intricate substructures of martensite for the hot-stamped (**d**,**e**) and bake-hardened (**f**) samples. (**g**) is EDX analysis of the precipitate in (**f**).

Hot dipping did not affect the phase transformation of the steel substrate, as its temperature was even lower than the continuous annealing temperature (Figure 1). That is to say, the microstructural evolution of the steel substrate should be similar for both processes. Figure 2 shows microstructure characters at different typical processing steps. The cold-rolled specimens had pancake-shaped pearlite (P) and ferrite (F) that were distributed alternatively on the cross section, and their elongation direction was parallel to the rolling direction, i.e., horizontal direction (Figure 2a). After continuous annealing, the microstructure exhibited annealed polygonal ferrite and degenerated pearlite, as shown in Figure 2b. This means that the deformed ferrite grains occurred to partial recrystallization behaviors, and the pearlite was degenerated into globular-like pearlite due to the annealing temperature a bit higher than the Ac1 temperature of this middle carbon steel. The full martensite (M) became the predominant microstructure after hot stamping (Figure 2c), but it is hard to identify their intricate substructures clearly under optical microscope.

Using TEM observation, numerous carbides appeared inside the martensitic laths for the hot-stamped specimen (as arrowed in bright field image of Figure 2d). According to our previous study [26], the die quenching had a much lower cooling rate than the water quenching, especially below the martensite start transformation temperature (~315 °C), and the auto-tempering induced carbon redistribution and agglomeration in the carbon-supersaturated ferritic matrix, resulting in the formation of numerous short rod-like cementites in a thermodynamically stable mode [27]. The dark field image in Figure 2e indicates that these carbides were preferentially distributed along the specific orientation of martensitic laths, e.g., habit planes. The bake hardening did not change the overall microstructure because of the very low tempering temperature. However, besides these tempering carbides, many nano-sized precipitates were present on the martensitic matrix (arrowed in Figure 2f). According to the EDX results (Figure 2g), they were composed of the elements Nb, V, Ti and C, which should be originated from the upstream heat treatments, e.g., hot rolling and hot stamping. Their presence is partly responsible for the strength improvement by precipitation strengthening to achieve 2 GPa, as reported in the literature [7,26].

**Figure 3:**
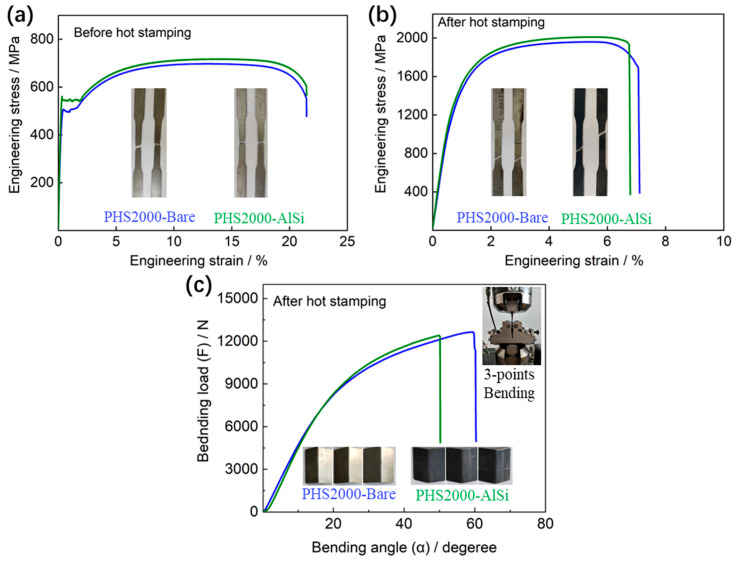
Tensile curves of the investigated PHS2000-Bare and PHS2000-AlSi steels before (**a**) and after (**b**) hot stamping steps and (**c**) the bending load-angle curves of the specimens in hot-stamped condition.

Figure 3a,b shows the engineering stress–strain curves of the investigated PHS2000-Bare and PHS2000-AlSi steels before and after the hot stamping steps. The result shows that the ultimate tensile strength and total elongation of two steels before hot stamping steps is almost the same, approximately 700 MPa. The yield strength of PHS2000-AlSi is slightly higher than that of PHS2000-Bare. After hot stamping, the ultimate tensile strength of PHS2000-Bare and PHS2000-AlSi steels is around 2000 MPa, with a total elongation of approximately 7%. The yield strengths of PHS2000-Bare and PHS2000-AlSi steels are 1395 MPa and 1428 MPa, respectively. However, the bending angle of PHS2000-Bare steel (59.1°) under the maximum bending load is greater than that of PHS2000-AlSi steel (49.8°), indicating that PHS2000-Bare steel has better toughness (Figure 3c).

### 3.2. Al-Si Coating Layer

**Figure 4:**
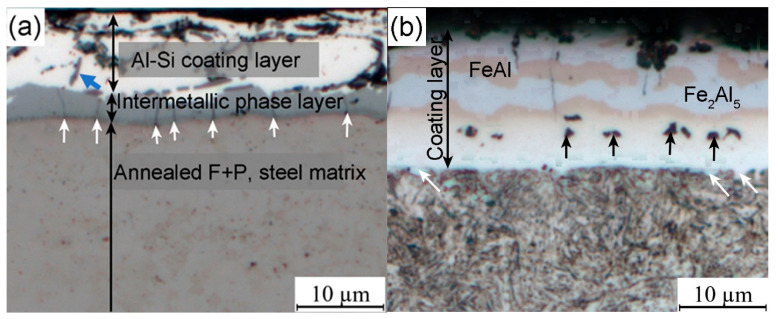
The morphology of Al-Si coating layer after the hot dipping (**a**) and hot stamping (**b**) steps.

**Figure 5:**
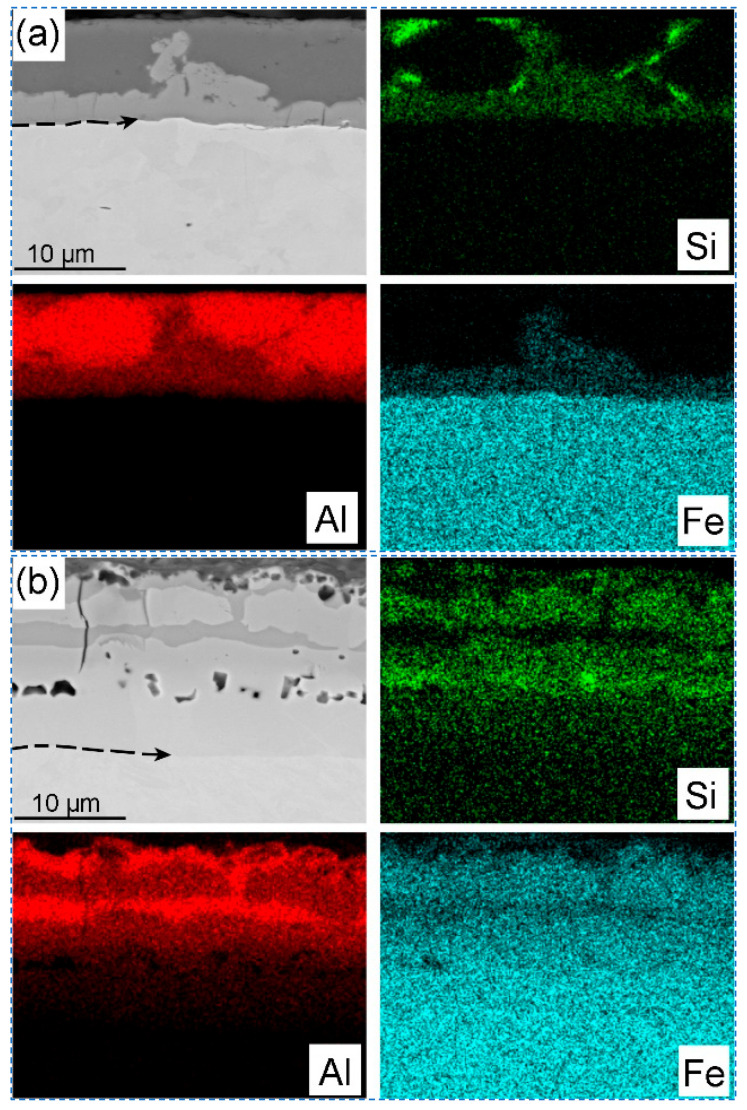
Distribution of chemical compositions at the cross-sectional coating layer: (**a**) hot-dipped condition; (**b**) hot-stamped condition.

**Figure 6:**
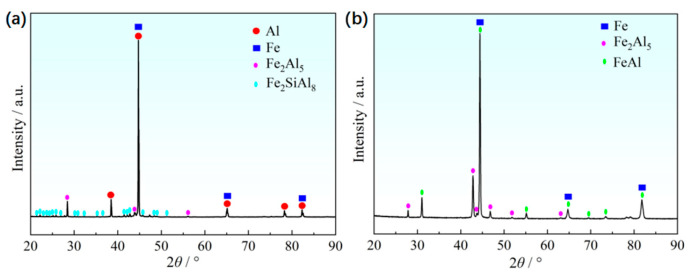
X-ray diffraction patterns of the Al-Si coatings: (**a**) hot-dipped condition; (**b**) hot-stamped condition.

Figure 4 shows the morphology of Al-Si coating layer before and after the hot stamping, and the SEM images with EDS mapping in Figure 5 represent the element’s distribution on the coating layer. Before the hot stamping process, the coating layer thickness of the hot-dipped samples was 10~14 μm, which actually includes two distinct sublayers (Figure 4a and Figure 5a). The outer layer was the solidification of the liquid Al-Si coating, which comprises irregular-shaped black phase and white matrix phase (Figure 4a). Based on the elements mapping, these randomly distributed black particles are high-Si phase (marked by blue arrow in Figure 4a) [28], while the matrix phase should be a hypoeutectic Al-Si composition. By contrast, the inner gray layer is directly attached to the steel substrate, and the thickness ranges from 2.5 to 6 μm, with the irregular interface front adjacent to the Al-based melt (Figure 4a). This layer is obvious on the SEM image (Figure 5a) and has clear boundaries compared to the steel substrate and Al-based coating because of the atomic contrast difference. The arrow line in Figure 5a depicts a portion of the steel/coating interface. The elements Fe, Al, and Si mappings indicate that this sublayer must be the intermetallic phase that was formed in this ternary alloy system. That is, the Fe has reacted with the melting Al-Si to form the new intermetallic phase during the short time of hot dipping. According to X-ray diffraction patterns of the Al-Si coatings in hot-dipped condition, the new phases formed at the hot dipping stage consisted of Fe_2_Al_5_ and Fe_2_SiAl_8_ (Figure 6a), and their type changed with the duration time due to intensive iron diffusion. This is consistent with the research results of Windmann and his colleagues [29,30]. Cheng and Wang [31] also identified them as hexagonal Al_7_Fe_2_Si based on the EBSD pattern. In addition, many microcracks are present on this sublayer and vertical to the interface (arrowed in Figure 4a) due to this Al-rich intermetallic phase having very high brittleness.

After the hot stamping, besides the martensite transformation for the steel substrate (Figure 2c), the coating layer also obviously changed at a relatively high temperature. The new phases of coating layer in hot-stamped conditions consisted of Fe_2_Al_5_ and FeAl (Figure 6b). The difference in the chemical potential of the elements Fe, Al, and Si between the coating layer and the steel substrate drives their intensive interdiffusion. As a result, their distribution is more homogeneous in the coating layer region compared to the former stage. Especially for Fe (Figure 5b), the coating layer has a quite similar concentration as in the steel substrate because of the higher diffusivity of Fe atoms in the liquid Al compared to the diffusion of aluminum in solid γ-Fe [29]. It is expected that some new intermetallic phases (e.g., FeAl, FeAl_3_, and Fe_2_Al_5_) could be formed based on the Fe-Al phase diagram [29]. Figure 4b shows the four sublayers’ morphology of the coating. Combined with the EDS mappings and the EBSD phase identification (given below), the top and third sublayers consist of the Fe_2_Al_5_ phase (light white area in Figure 4b), while the other two sublayers are the FeAl phase (light brown). The Si presence to some extent influences the distribution of these intermetallic phases, as shown in the Si mapping (Figure 5b) that the Si is relatively enriched on the FeAl phase, because this phase has higher solubility for Si than the Fe_2_Al_5_ phase.

When the Al-Si coating in the hot-dipped condition is heated at the austenitizing temperature, the Fe_2_Al_5_ phase located at the interface of the steel substrate transforms into the FeAl phase, mainly due to the diffusion of Fe into the coating and Al into the steel substrate. The highest Fe content in the coating is found directly at the interface to the steel substrate. Thus, the phase of type FeAl is first formed at this interface, and the layer grows towards the Al-Si coatings as the austenitization time increases [28,29]. Due to the low solubility of Si in the Fe_2_Al_5_ phase, Fe_3_Al_2_Si_3_ forms at the grain boundaries of the Fe_2_Al_5_ phase during the austenitization process. Due to the diffusion of Fe, the Al-rich phase Fe_2_SiAl_8_ transforms into the Fe-rich Fe_2_Al_5_ phase during the austenitization process. The growth kinetics of Fe_2_Al_5_ are stronger than that of Fe_2_SiAl_8_ and Fe_3_Al_2_Si_3_ [29], so it occupies the largest volume proportion in the Al-Si coating. The formation mechanism of another type of FeAl phase is as follows: With the progress of austenitization, the Si in Fe_3_Al_2_Si_3_ diffuses into the steel substrate. When the Si content in Fe_3_Al_2_Si_3_ is below the critical value, it will transform into a more stable FeAl phase. Due to the formation of Fe_3_Al_2_Si_3_ at the initial Fe_2_Al_5_ phase grain boundary, this type of FeAl phase is distributed in a band-shaped morphology in the Fe_2_Al_5_ phase.

Meanwhile, many micro-voids (signified with black arrows in Figure 4b) can be observed at the bottom layer of the coating, which are known as Kirkendall voids due to the difference in the diffusion rate of the elements between the steel substrate and the Al-based coating [29]. Therefore, the inner border of the Kirkendall voids is normally considered to be the original Al coating/steel substrate interface [32]. However, these voids always distribute heterogeneously for industrial steel sheets and their formation depends on the soaking time [29]. On account of the diffusion of Al into the steel substrate, the Al-rich ferrite layer could be formed in the vicinity of the interface adjacent to the steel substrate, and some fine ferrite grains have already grown into the steel matrix, as marked with white arrows in Figure 4b. However, both the Al-rich ferrite layer and the FeAl phase have similar morphology and the same BCC crystal structure (given below), leading to the fact that they are not easy to take apart. To measure the coating layer thickness conveniently in industrial practice, here, we simply considered that the Al-rich ferrite layer is a part of the coating layer.

**Figure 7:**
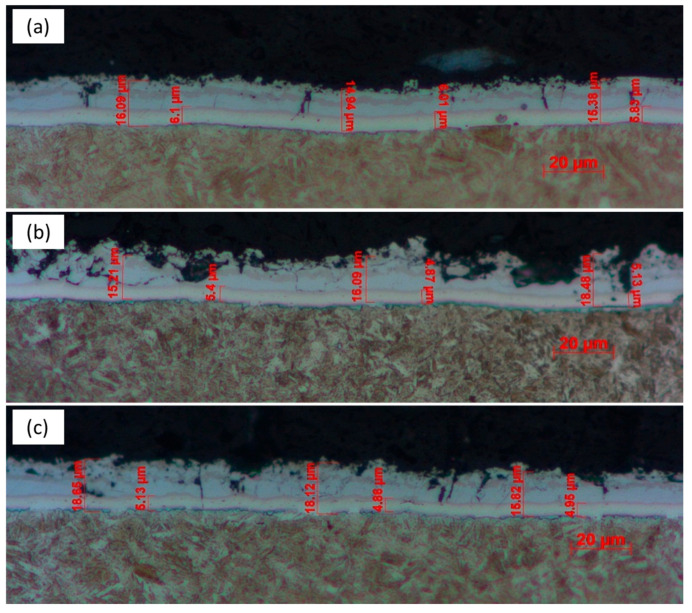
Statistical analysis of the coating layer thickness after the hot-stamped blanks were extracted from different locations of industrial steel coil: (**a**) the head of the coil, (**b**) the middle of the coil, and (**c**) the tail of the coil.

Figure 7 presents the Al-Si coating layer morphology of hot-stamped sheets that were extracted from different positions of the industrial production steel coil. The coating layer/steel interface appears clearly after the sample etching. It can be seen that the cross-sectional outline of the coating layer is not quite uniform, especially in the middle section of the steel coil that the outer layers sometimes disappear and sometimes become thicker, which makes the coating layer surface ragged. The entire thickness of coating layer was measured to be ~ 17 μm, excluding those areas with an unusual outline. By contrast, the bottom sublayer (including the newly formed Al-rich ferrite layer) is very uniform, and the main range of thickness is about 4.8~6.1 μm. Due to the presence of the brittle Fe2Al5 phase, the microcracks can be frequently observed at the outer layers of the coating, but they are always terminated at the interface of the bottom FeAl sublayer. These pre-existing microcracks will deteriorate the bending property as well as the corrosion resistance [23]. In this sense, updating the current industrial processing parameters is necessary to avoid the appearance of a brittle phase in the Al-based coating layer. For example, appropriately extending the soaking time can promote the transition of brittle Fe_2_Al_5_ to the ductile FeAl phase [29].

### 3.3. Crystallography

**Figure 8:**
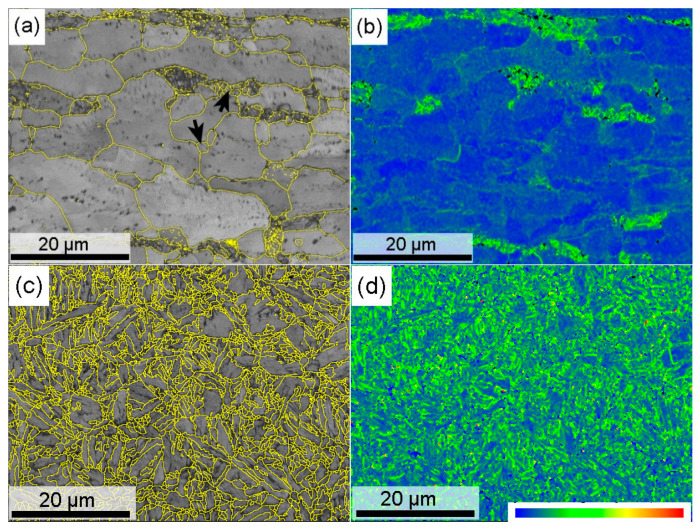
EBSD results of steel substrate at different processing steps (**a**,**b**) hot dipping, (**c**,**d**) hot stamping, (**a**,**c**) image quality maps superimposed with misorientation angle boundaries (yellow lines) higher than 10 deg, (**b**,**d**) KAM maps showing the distribution of local misorientation.

Figure 8 shows the EBSD results of the continuously annealed and hot-stamped microstructures at the steel substrate. The image quality map (Figure 8a) exhibits two distinct band contrast domains. Combined with the microstructure in Figure 2b, the dark gray domains imply that they have the lower band contrast, which must be the degenerated pearlite, since the phase interfaces inside the pearlite lowers the quality of Kikuchi pattern. The bright gray domains correspond to the annealed ferrite, including prior elongated coarse grains and newly recrystallized fine grains (arrowed). All these grains have grain boundaries with high misorientation angle (>10°), as delineated with yellow lines. Kernel average misorientation (KAM) maps, which were produced by a 5-pixel by 5-pixel filter with boundaries of misorientations greater than 5 ° excluded, represent the distribution of geometrically necessary dislocations [33]. It is obvious that the degenerated pearlite domains have higher local misorientation than the ferrite grains. For individual ferrite grains, the greater the pancake shape remains, the higher the degree of local misorientation, which means the present intercritical annealing cannot eliminate the deformation dislocations completely.

After the hot stamping, the high density of high misorientation boundaries distribute evenly on the matrix (Figure 8c), as the martensite transformation produces many block and packet boundaries with a higher misorientation angle in each prior austenite grain, which refines the effective grain size. Meanwhile, high density dislocations were induced to accommodate the intensive transformation strain during martensite transformation, as presented by the KAM map in Figure 8d. These features should be responsible for the hot-stamped sample having an ultra-high strength. On the other hand, they also deteriorate the resistance to hydrogen embrittlement, because the interaction of hydrogen and mobile dislocations acts as a root to promote the hydrogen-induced cracking [23]. In a sense, the hydrogen also threatens the delayed fracture of industrial products if a certain hydrogen is present, which implies that the hydrogen evolution needs to be clarified for every industrial production line to protect their products from delayed fracture.

**Figure 9:**
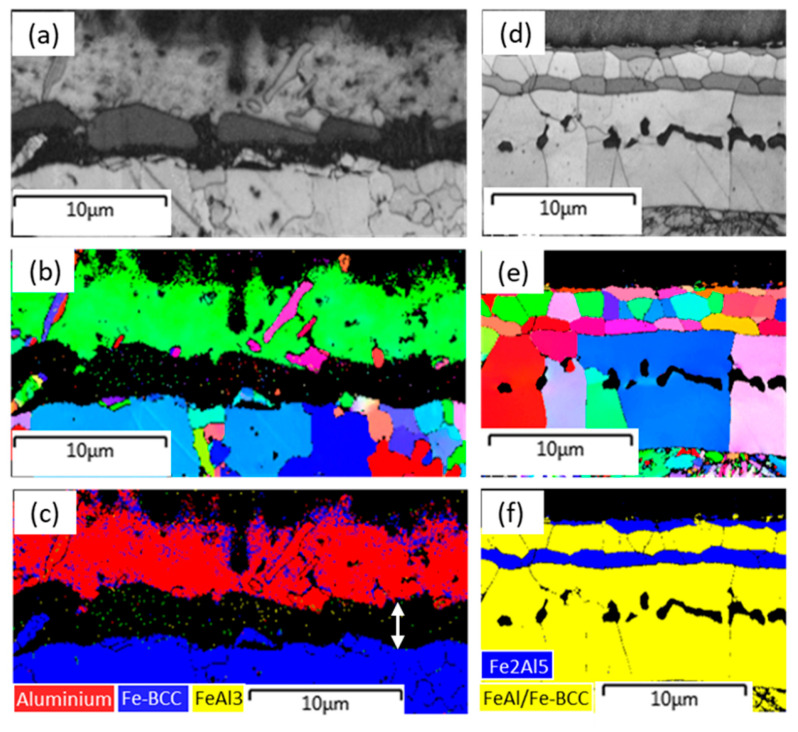
Crystallographic features of the coating layer at different processing steps: (**a**–**c**) hot dipping, (**d**–**f**) hot stamping, (**a**,**d**) image quality map, (**b**,**e**) orientation map, and (**c**,**f**) phase distribution map.

The crystallographic features of the coating layers before and after hot stamping are revealed in Figure 9. Before the hot stamping, the outer coating layer was identified as a predominantly Al-based single crystal structure, and only a few of the fine Al-based grains with different orientations randomly distribute on this matrix orientation (Figure 9a,b). In this study, the intermetallic layer between the Al-based coating and the steel substrate (double arrow in Figure 9c) was not identified due to lack of the crystal data of this intermetallic phase in our EBSD software-Attec^®^ 5.0 SP1. As mentioned above, this phase should be Al8Fe2Si, and nearly no FeAl3 phase exists at the hot dipping stage.

After hot stamping, it is clear that the coating layer contains four sublayers, some of which have their own unique feature for crystallographic grains, which makes all sublayer boundaries very clear (Figure 9d,e). In general, all grains at each sublayer have a character of random orientation. Based on the phase identification (Figure 9e), the outermost and third sublayers are the Fe_2_Al_5_ phase with similar pancake-shaped grains. The second and bottom sublayers are the FeAl phase (or BCC type crystal), but their grain size is quite different. Note that the martensite at the bottom of Figure 9e is also depicted in yellow because of the same BCC crystal structure. In the crystallography, the fourth sublayer exhibits typical columnar grains, as the grain boundaries are normally perpendicular to the interface boundary and cross the entire thickness. This sublayer contains two typical compositions of Fe-rich intermetallic phase and Al-rich alpha ferrite that are largely separated by the inner border of Kirkendall voids (Figure 5b). Nevertheless, these individual columnar grains have an extremely uniform orientation, indicating that the Fe-rich intermetallic phase occurs at the epitaxial growth attached to the Al-rich ferrite grains. The growth of Al-rich ferrite is controlled by the diffusion of Al in γ-Fe; thus, the growth rate constant estimated is about 0.337 μm s^−1/2^ based on the parabolic diffusion-controlled growth law ($D=k\times \sqrt{t}$, where *D* is the thickness of the reaction phase, *t* is the holding time, *k* is the temperature-dependent rate constant). Although this value is nearly twice as high as the results (~0.185 μm s^−1/2^) by Springer et al. [34], who studied the growth rate of total Fe-Al reaction layer thickness at the lower temperature of 600 °C, this difference is acceptable due to its temperature dependence (the present soaking temperature was as high as 925 °C).

The main purpose of Al-based pre-coating is to prevent the strong oxidation of the steel sheets during austenitization at a high temperature. On the other hand, the presence of the coating layer also has some extra effects on the metallurgical behaviors and final mechanical properties. As mentioned above [17,18,19,20], the Al-Si coating layer has promoted the hydrogen uptake during hot stamping simulations. In combination with actual industrial production, the hydrogen evolution with and without hot dipping was comparatively explored below.

### 3.4. Hydrogen Evolution

**Figure 10:**
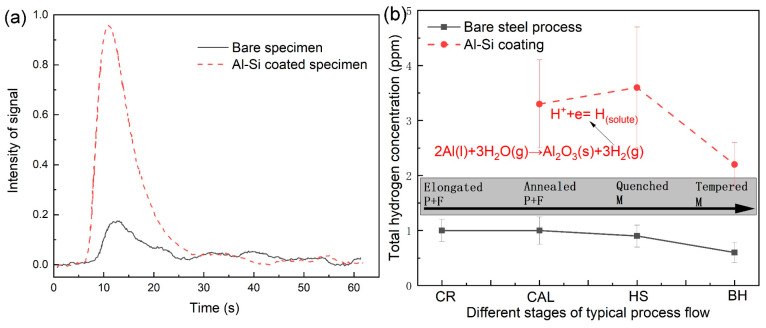
Hydrogen signal curves of two hot-formed specimens with or without an Al-Si coating (**a**) and the evolution of hydrogen concentration with two typical production flows (**b**).

The curves of the hydrogen signal intensity of the bare steel and Al-Si-coated specimens at the hot stamping stage are exemplified in Figure 10a. Obviously, the Al-Si-coated specimen has a significantly stronger hydrogen signal than the bare steel specimen. The total hydrogen content was determined based on the integral of these signal curves and calibration using standard Leco specimens.

Figure 10b summaries the total hydrogen content as a function of process steps for these industrial production flows, accompanied with the microstructural evolution. Note that it can be only regarded as semi-quantitative results because of the limits of resolution for the hydrogen analysis. For the bare steel process, the total hydrogen content is always kept at a very low level, normally lower than 1 ppm. By contrast, the hydrogen concentration for the Al-Si-coated specimens notably increased after hot-dip aluminizing, which is different from the reports in the literature [14]. Subsequently, the hydrogen content further increased after hot stamping, which should be attributed to the fact that molten Al-Si coating layer reacts with moisture (dewing points) during austenitizing (as given by the chemical equation in Figure 10b), producing hydrogen atoms that intrude into the steel substrate, since the diffusion coefficient of hydrogen in the molten Al-Si coating is very high (nearly 1 μm^2^/s). At this moment, the microstructure at the steel substrate was fully austenitic, which has a higher solubility of hydrogen, leading to the net diffusion flow of hydrogen from liquid Al-based coating to the steel. These intruded hydrogen atoms will be trapped in the steel substrate after fast cooling due to the solidified coating layer preventing the hydrogen out-diffusion. This prevention is originated from the decrease in hydrogen diffusion coefficient in the coatings, as reported by Krid et al. [20], because of a denser packing structure of the Fe_2_Al_5_ and the crystal lattice expansion and chemical trapping of hydrogen near the substitute elements, i.e., Si and Al. This is in good agreement with the results of hydrogen generation and uptake of the aluminized steel during laboratory-simulated hot stamping [14,15,17,18,19,20].

Similarly, the reason that relatively higher hydrogen content exists in the continuously annealed and hot-dip aluminized specimen is due to the presence of a hydrogen gas component in protective gas (i.e., N_2_ + 20% H_2_). The gaseous hydrogen can be absorbed on the surface of the steel sheet during continuous annealing. Actually, the gaseous hydrogen charging was usually conducted at a relatively higher temperature in order to accelerate the hydrogen permeation [35]. A portion of the surface-absorbed hydrogen would be sealed by hot-dip aluminizing, because the hot-dip aluminizing was tightly followed by the continuous annealing (their interval time was less than 1 s), and the hot-dip process was completed in several seconds. By contrast, for the bare steel process, the annealed specimen surface might also have some absorbed hydrogen atoms, but they can escape quickly during cooling in air due to no obstacles of hydrogen degassing. That is the reason why the hot-dip aluminized specimen increased the hydrogen content notably compared to the specimen without the aluminizing process.

These hydrogen atoms that were sealed by a hot-dip-coated layer can be partly inherited to the hot stamping step or maybe degassed out during the reheating stage of the hot stamping. It is hard to determine whether the hydrogen atoms can be inherited between these two heat treatment steps. Valentini et al. [21] demonstrated that the higher the initial hydrogen content, the higher the concentration of hydrogen can be obtained after hot stamping. This implies that the hydrogen in the hot-dipped samples can be partially retained to the hot-stamped specimens.

When the process moved to the bake hardening step, the hydrogen content decreased due to the hydrogen degassing for both processes. Irrespective of this, the Al-Si-coated specimens still have a relatively higher hydrogen content than the bare steel specimens, meaning that the coated layer inhibited the hydrogen out-diffusion during bake hardening process, and a portion of hydrogen that was produced during the former processing steps will be inherited into the products if the Al-Si-coated layer was present.

In combination with microstructural evolution, although the hydrogen content was increased at continuous annealing plus the hot-dip aluminizing step, it is less detrimental to the HE resistance of the steel sheet, since the degenerated pearlite and ferrite formed at this stage are not highly susceptible to the HE, and these hydrogen atoms can be partly degassed out at the next heat-treated step. However, hydrogen atoms that formed at the hot stamping stage due to the presence of Al-based layer mostly threaten the HE resistance of ultra-high-strength steels, because the martensite with high HE susceptibility forms simultaneously, and these hydrogen atoms can be largely inherited into the final products due to no heat treatments after bake hardening. Thus, the hydrogen-assisted delayed fracture of the Al-Si-coated steel sheets should be checked in future study, and the dehydrogenation process may be figured out to make their industrial application acceptable.

Based on the above analysis, strictly controlling the dew point of the furnace in the hot stamping process may be an effective measure to avoid the hydrogen embrittlement of PHS. Furthermore, based on the hydrogen evolution curves observed during various processing stages, low-temperature tempering (170 °C for 20 min) promotes hydrogen diffusion in hot-formed specimens whether Al-Si-coated or uncoated, resulting in reduced hydrogen content and consequently achieving a synchronous improvement in the hydrogen embrittlement resistance of PHS. Therefore, low-temperature tempering is also an effective measure to reduce the risk of the hydrogen embrittlement of PHS.

This study focuses on microstructural evolution and the internal hydrogen content of ultra-high-strength automotive steels during two typical industrial production flows, providing reference for the smooth industrial production of PHS production and ensuring the manufacturing of qualified automotive parts. Sufficient statistical data can indeed play a good supporting role in the conclusion. We did conduct the statistical analysis on the significant testing, such as hydrogen content determination. However, as mentioned above, the distribution and diffusion of hydrogen in steel are affected by many factors, and the detection method used in this paper has certain limitations, so the uncertainty was noticeable for every processing step. In the future, quantitative analysis, such as phase abundance, hydrogen embrittlement mechanism, and critical values leading to hydrogen embrittlement will be the focus of our research, including the detection and analysis of diffused hydrogen and non-diffusible hydrogen content using the equipment of thermal desorption spectroscopy (TDS).

## 4. Conclusions

Two typical industrial production flows of ultra-high-strength automotive steel were investigated to comparatively reveal their microstructural evolution (including Al-Si coating layers) and hydrogen content with the progress of the processing steps. The main findings are given below.

(1) The steel substrate showed similar microstructural evolution with the production steps from cold rolling to bake hardening: pancake-shaped pearlite and ferrite, degenerated pearlite and annealed ferrite, full lath martensite, and then tempered martensite. Micro-alloying carbides can be retained in the final microstructure, which should play a role in optimizing the mechanical properties.
(2) The Al-Si coating layer became thicker due to the newly formed Al-rich α-ferrite after being subjected to hot stamping; although, the entire thickness was not uniform for the present industrial production steel coil. The constituent phases of the coating layer were changed into the multi-layer structure of FeAl and Fe_2_Al_5_, and many microcracks were found to be across the brittle phase Fe_2_Al_5_. The pre-existing microcracks and higher hydrogen content may be detrimental to the bending property.
(3) Compared with the bare steel process, the Al-Si-coated specimens always have a higher hydrogen content. That is not only because of the molten Al reaction with moisture during the heating process in the hot stamping stage but also due to the surface absorption in the hydrogen containing a protective atmosphere during the hot dipping process.

## Data Availability

The original contributions presented in this study are included in the article. Further inquiries can be directed to the corresponding authors.
